# Molecular and Phenotypic Investigation on Antibacterial Activities of Limonene Isomers and Its Oxidation Derivative against *Xanthomonas oryzae* pv. *oryzae*

**DOI:** 10.4014/jmb.2311.11016

**Published:** 2024-01-17

**Authors:** Hyeonbin Kim, Mi Hee Kim, Ui-Lim Choi, Moon-Soo Chung, Chul-Ho Yun, Youngkun Shim, Jaejun Oh, Sungbeom Lee, Gun Woong Lee

**Affiliations:** 1 Green-Bio Division, Jeonju AgroBio-Materials Institute, Jeonju 54810, Republic of Korea; 2 Division of Radiation Biotechnology, Advanced Radiation Technology Institute, Korea Atomic Energy Research Institute, Jeongeup 56212, Republic of Korea; 3 School of Biological Sciences and Technology, Chonnam National University, Gwangju 61186, Republic of Korea; 4 Microzyme Co., Ltd. Research and Development Department, Damyang-gun, Jeollanam-do 57385, Republic of Korea; 5 Department of Radiation Science and Technology, University of Science and Technology, Daejeon 34113, Republic of Korea

**Keywords:** *Xanthomonas oryzae* pv. *oryzae*, limonene, carveol, antibacterial activity, virulence, biofilm

## Abstract

*Xanthomonas oryzae* pv. *oryzae* (*Xoo*) causes a devastating bacterial leaf blight in rice. Here, the antimicrobial effects of D-limonene, L-limonene, and its oxidative derivative carveol against *Xoo* were investigated. We revealed that carveol treatment at ≥ 0.1 mM in liquid culture resulted in significant decrease in *Xoo* growth rate (> 40%) in a concentration-dependent manner, and over 1 mM, no growth was observed. The treatment with D-limonene and L-limonene also inhibited the *Xoo* growth but to a lesser extent compared to carveol. These results were further elaborated with the assays of motility, biofilm formation and xanthomonadin production. The carveol treatment over 1 mM caused no motilities, basal level of biofilm formation (< 10%), and significantly reduced xanthomonadin production. The biofilm formation after the treatment with two limonene isomers was decreased in a concentration-dependent manner, but the degree of the effect was not comparable to carveol. In addition, there was negligible effect on the xanthomonadin production mediated by the treatment of two limonene isomers. Field emission-scanning electron microscope (FE-SEM) unveiled that all three compounds used in this study cause severe ultrastructural morphological changes in *Xoo* cells, showing shrinking, shriveling, and holes on their surface. Moreover, quantitative real-time PCR revealed that carveol and D-limonene treatment significantly down-regulated the expression levels of genes involved in virulence and biofilm formation of *Xoo*, but not with L-limonene. Together, we suggest that limonenes and carveol will be the candidates of interest in the development of biological pesticides.

## Introduction

The gram-negative bacteria *Xanthomonas oryzae* pv. *oryzae* (*Xoo*) causes a bacterial leaf blight in rice plants [1]. It is a devastating disease in rice, which reduces grain yield up to 30% when the damage is serious. *Xoo* enters into leaf tissues through wounds or hydathodes of rice plants and migrates to the xylem to proliferate [2].

*Xoo* causes virulence through enzymatic and non-enzymatic mechanisms [3]. For examples, biofilm is one of the most common virulence factors of various microorganisms and helps bacteria adhere to each other [4, 5]. This biofilm also contributes to the attachment of the bacteria to xylem [6]. Therefore inhibiting the formation of biofilm has become a strategy to control pathogenic microorganisms [7]. Pectinase, an extracellular enzyme, induces host defense responses [8]. Xanthan of a large amount of exopolysaccharide (EPS) from *Xanthomonas* spp. is a well-known virulence factor [9, 10]. The type III secretion system causes cell death at the plant infection site [11, 12]. Quorum sensing (QS) causes coordinated biofilm formation and increases virulence through motility, chemotaxis, of stress response, and synthesis EPS and extracellular enzymes [13-15].

Terpenoids are most abundant compounds among plant secondary metabolites. Limonene is a cyclic monoterpene (C10) found mainly in the oil of citrus fruit peels and D-isomer is commonly found in nature as a fragrance [16]. In our previous study, we revealed that abiotic stresses such as hydrogen peroxide, UV-B radiation, and γ-irradiation strongly induced the production of volatile monoterpene limonene in rice seedlings. We further confirmed that the limonene was able to strongly suppress the growth of *Xoo* in a disc diffusion assay and in vivo infection assay [17]. Interestingly, the degree of antimicrobial activity was dependent on a chirality of the compounds of which (S)-limonene showed higher antimicrobial activity than that of (R)-enantiomer.

It is known that carveol, a limonene oxidation derivative, has antibacterial activity against *Staphylococcus aureus*, *Salmonella enterica* serovar Typhimurium, *Escherichia coli*, and *Bacillus cereus* using microdilution method [18]. D-Limonene was found to have antibacterial activity against *Salmonella senftenberg*, *E. coli*, *S. aureus*, and *Pseudomonas* species by comparing growth inhibition rates in nutrient broth [19]. It also has an antifungal effect through weakening the cell wall of *Saccharomyces cerevisiae* [20]. Carveol and D-limonene have been actively studied to evaluate their antibacterial activity against human pathogens, but the research with plant pathogens has been limited. Moreover, little is known about differential effect derived from isomeric properties of limonenes (*e.g.*, D-limonene and L-limonene) on the antimicrobial activity.

In this study, we report the inhibitory effect of limonene isomers and its oxidation derivative carveol on the growth of *Xoo* in a dose-dependent manner. The mechanism of the inhibition was investigated by performing crystal violet assay, motility assay, and xanthomonadin quantification. A field emission-scanning electron microscopy revealed that the monoterpenoids cause severe morphological changes of *Xoo*. Further, we confirmed the differential expression of the genes involved in the virulence and biofilm formation of *Xoo* with the treatment of the terpenoids.

## Materials and Methods

### Measurement of Inhibition Effect in Liquid Cultures

*Xoo* culture (OD_600_ = 1.0, 2 ml) was inoculated into 10 ml of nutrient broth (NB) (BD Difco, USA) with or without a compound in various concentrations (0.1, 0.4, 0.7, 1, 2, 4, 6, 8, or 10 mM). After incubation for 48 h in a shaking incubator at 28°C, OD_600_ was measured and growth rates were compared for each compound concentration.

### Evaluation of Motility

Motility of *Xoo* was evaluated after the treatment of various concentrations of a compound through swarming and swimming characteristics. Each compound dissolved in DMSO was added to sterilized swarming (NB, 0.5%agar) and swimming media (NB, 0.3% agar), respectively, and solidified in a petri-dish. *Xoo* culture (5 μl droplet) was added on each plate and incubated for 7 days at 28°C. Each plate was evaluated for swimming and swarming by measuring the diameter of the bacterial zone.

### Detection of Biofilm Using Crystal Violet Assay

Crystal violet assay was performed to detect the biofilm of *Xoo* grown using brain heart infusion (BHI) broth (BD Difco) supplemented with 5% (w/v) sucrose. Each compound in various concentrations was added to the medium and 100 μl aliquot was transferred into each well of a 96-well cell culture plate. Then, 20 μl of *Xoo* culture was inoculated into the wells and incubated without shaking for 7 days at 28°C. After the incubation, each well was washed three times using deionized water (DIW) and stained with 200 μl of 0.1% (w/v) crystal violet solution for 15 min. Sequentially, each well was washed three times using DIW and air dried. Crystal violet was dissolved with 100% ethanol for 15 min and biofilm was measured at OD_590_.

### Estimation of Extracted Xanthomonadin

Cell pellets collected from 4 ml of *Xoo* suspension with or without a compound was mixed with 1 ml 100%methanol. The mixture was further incubated in darkness for 10 min on a rotating shaker and the cells were collected by centrifugation at 12,000 g for 8 min. The xanthomonadin pigment from the collected supernatant was measured at OD_445_ [21, 22].

### Field Emission-Scanning Electron Microscope (FE-SEM) Observation

*Xoo* was incubated in NB medium with or without a compound at 28°C shaking incubator for 12 h. The cultures were centrifuged and suspended in 2% paraformaldehyde and 2% glutaraldehyde in 0.05 M sodium carcodylate buffer (pH 7.2). The suspensions were incubated (pre-fixation) at 4°C overnight and then were washed three times with 0.05 M sodium carcodylate buffer (pH 7.2). The suspension was further incubated (post-fixation) for 1.5 h in 1% osmium tetroxide. Then, the suspension was dehydrated by ethanol gradient ranging from 30% to 100% for 3 min incubation. After the dehydration, hexamethyldisilazane (100%) was added and the cells were dried at room temperature. Images were taken at magnifications of × 10,000 and × 40,000 using SUPRA-40VP (Zeiss Germany) equipment.

### Real-Time PCR

Gene specific primers for virulence and biofilm formation were designed using Primer 3 (version 0.4.0) templating the gene sequences available in the NCBI (Table 1). *Xoo* cultures incubated NB medium with or without a compound were lysed using TRI reagent (Molecular Research Center, Inc., USA). The RNA was isolated with chloroform and precipitated with isopropanol. RNeasy mini kit (Qiagen, USA) was used to wash column-based isolated RNA. During washing, genomic DNA was removed with RNase-Free DNase Set (Qiagen). For cDNA synthesis, the RT series (Biofact, Republic of Korea) was performed according to the manufacturer’s procedure. Real-time PCR was performed with 2X Real-Time PCR Master Mix (Biofact) according to the manufacturer’s instructions. The PCR cycle consisted of the following steps: 15 minutes at 95°C and 40 cycles of 20 s at 95°C and 30 s at 58°C. The expression level of 16S rRNA was used to determine the relative expression level of target genes. The relative expression level of each gene was calculated by 2^-ΔΔCt^ method [23].

### Statistical Analysis

Each experiment was performed in three technical replicates and three biological replicates. The results obtained from each experiment were expressed as mean and standard deviation (SD) in triplicate. The significance of the control group and each experimental group was compared using the Dunnett test after analysis of variance after one-way ANOVA at the *p* < 0.05 level (*p < 0.05, **p < 0.01, and ***p < 0.001). Statistical analysis was performed using GraphPad Prism5 (ver.5.0, USA).

## Results

### Measurement of Inhibition Effect in Liquid Cultures

**Fig. 1:**
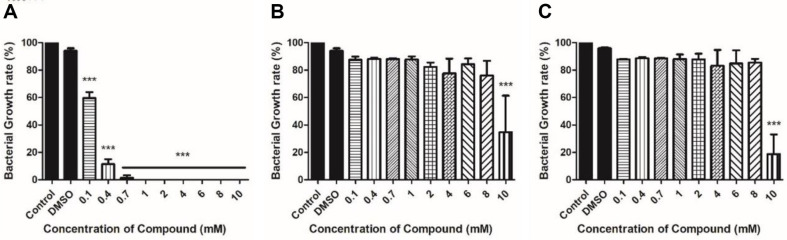
Measurement of bacterial growth rate calculated with OD_600_ of *Xoo* incubated in NB media treated with or without compounds for 48 h. (**A**) Carveol, (**B**) D-Limonene, and (**C**) L-Limonene.

It was confirmed that the growth of *Xoo* treated with carveol was significantly inhibited at 0.1 mM, and no growth was observed at ≥ 1 mM (Fig. 1A). However, D-limonene and L-limonene treatments cause the significant growth inhibition at as high as 10 mM (Fig. 1B and 1C).

### Evaluation of Motility

**Fig. 2:**
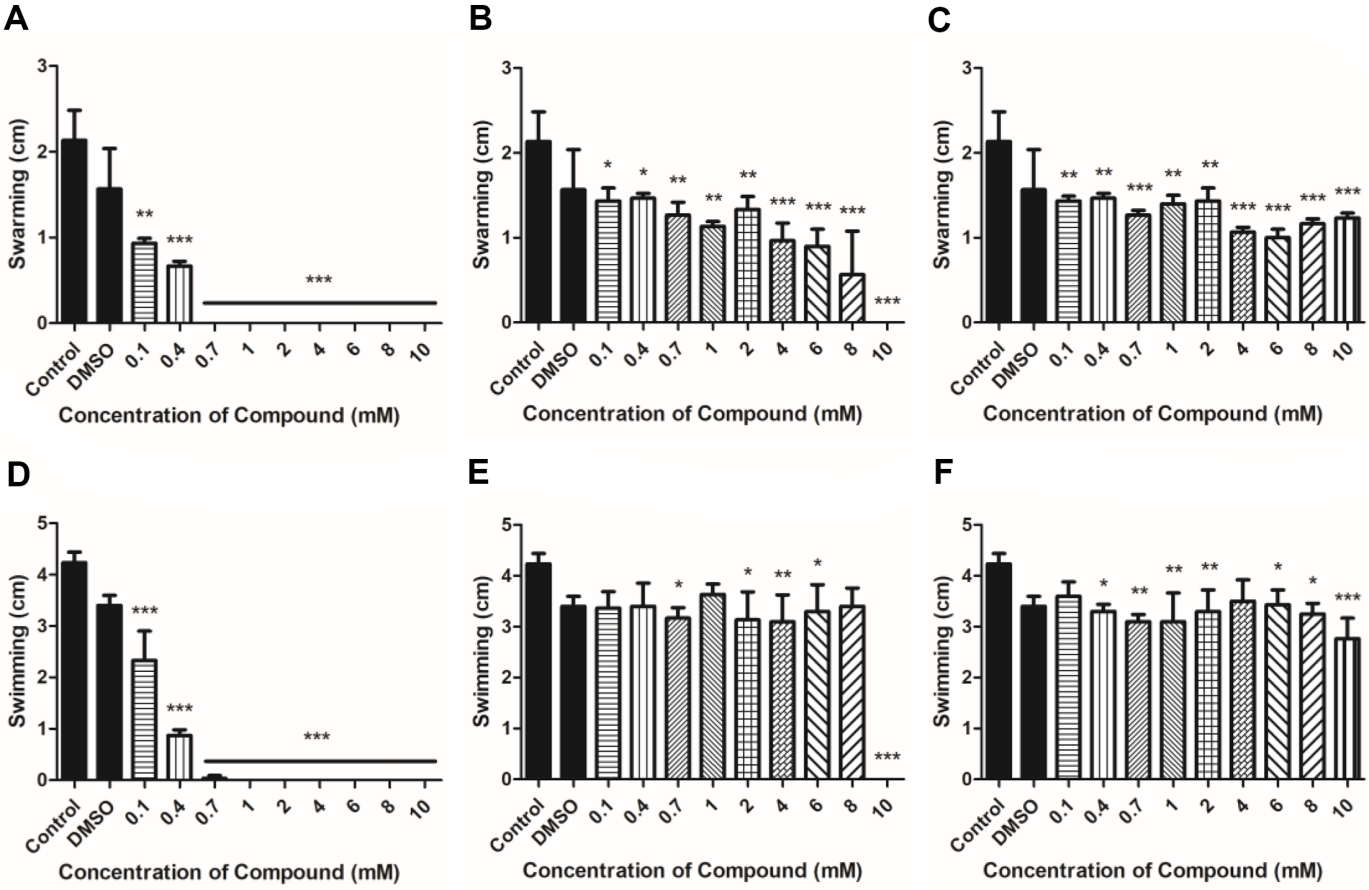
Result of motility evaluation through swarming and swimming level by measuring diameter of bacterial zone grown on swarming (NB, 0.5% agar) and swimming (NB, 0.3% agar) media added compounds. (**A-C**) Swarming analysis result, sequentially carveol, D-limonene, and L-limonene, (**D-F**) Swimming analysis result, sequentially carveol, D-limonene, and L-limonene.

As a result of swarming, it was confirmed that the bacterial zone of *Xoo* treated with carveol was inhibited starting from 0.1 mM and *Xoo* did not grow starting at ≥ 0.7 mM (Fig. 2A). Swimming results were also similar to those of swarming results (Fig. 2D). D-Limonene seemed to reduce the zone area in a concentration-dependent manner in the swarming analysis, and no growth of *Xoo* was observed at 10 mM (Fig. 2B). On the other hand, in the swimming results, there was negligible inhibition effect at 0.1 mM up to 8 mM, but with complete suppression at 10 mM (Fig. 2E). For L-limonene, the zone of motility of *Xoo* was smaller than that of *Xoo* without the compound in both swarming and swimming results, but the level of the inhibitory activity was lower when compared to that of D-limonene representing considerable *Xoo* growth even at 10 mM treatment (Fig. 2C and 2F).

### Detection of Biofilm using Crystal Violet Assay

**Fig. 3:**
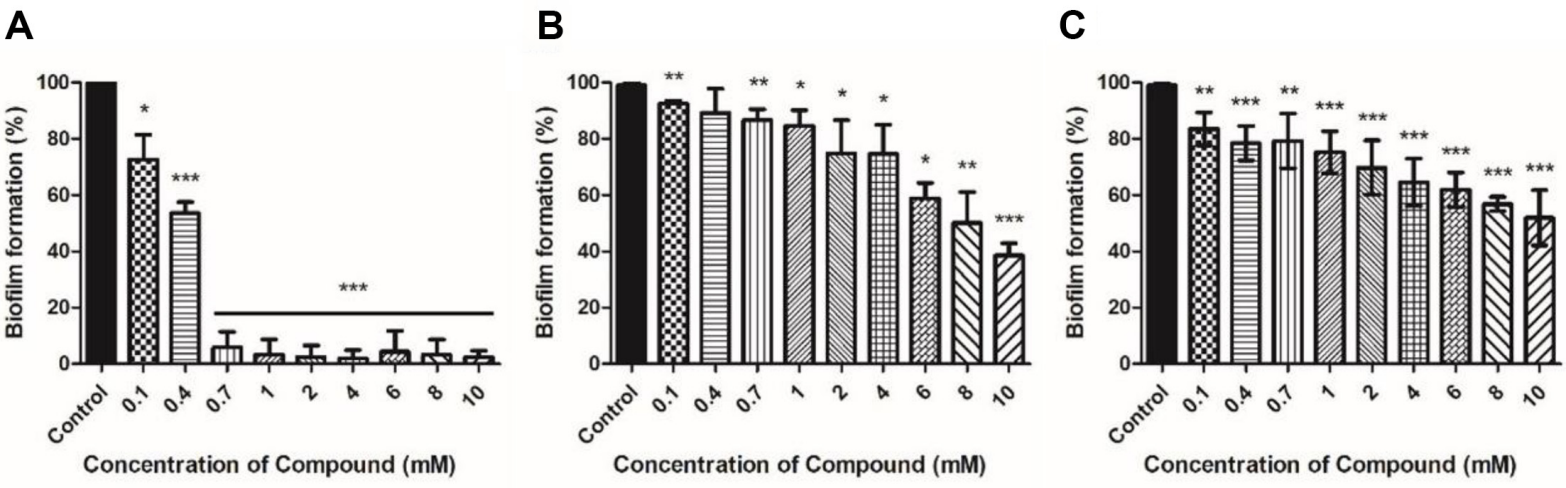
Comparative analysis of biofilm formation rate by measuring OD_590_ from a result of crystal violet assay after 7 days incubation without (control) or with treatment of various concentrations of each compound. (**A**) Carveol, (**B**) D-Limonene, and (**C**) L-Limonene.

*Xoo* culture treated with carveol at different concentrations showed a tendency to decrease biofilm production at 0.1 mM (≥ 20% suppression) to 0.4 mM (≥ 50% suppression) compared to that of the control (Fig. 3A). With the treatment of ≥ 0.7 mM carveol, the biofilm production was dramatically reduced (≥ 90% suppression). D-Limonene and L-limonene treatments also reduced biofilm formation of *Xoo* in a concentration-dependent manner, but the degree of effect was lower than that of carveol. D-Limonene and L-limonene treatments with as high as 10 mM still showed ≈ 40% of biofilm formation in comparison with that of control (Fig. 3B and 3C).

### Estimation of Extracted Xanthomonadin

**Fig. 4:**
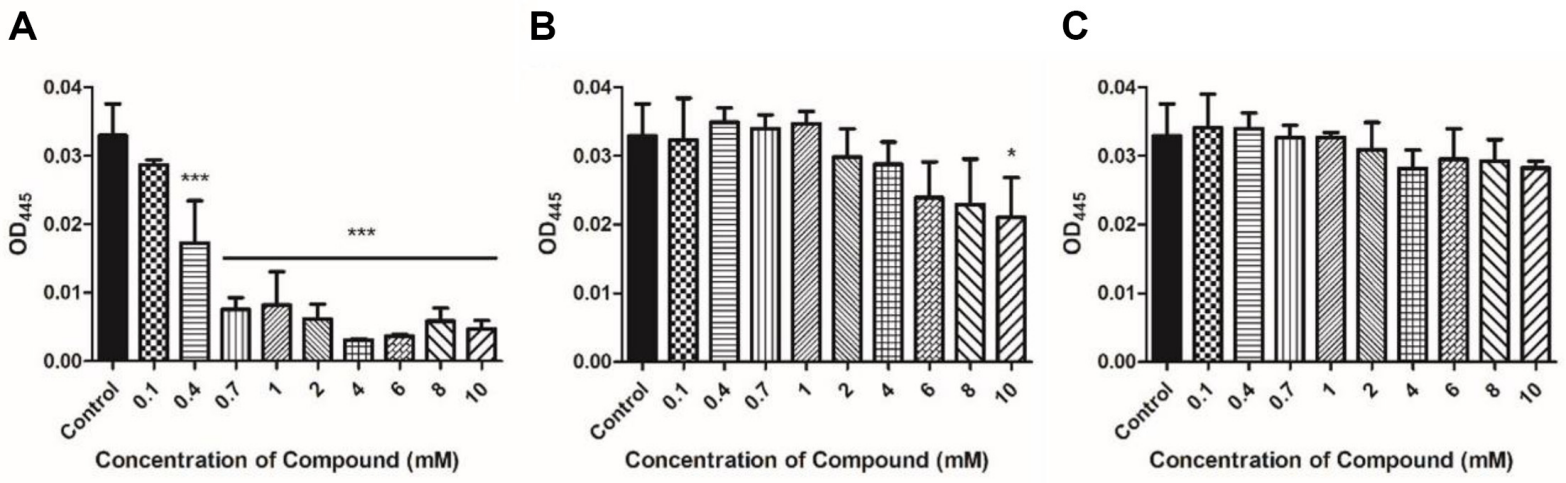
Estimation of extracted xanthomonadin by analyzing OD_445_ resulting from reacting cell pellet of *Xoo* treated compounds with 100% methanol. (**A**) Carveol, (**B**) D-Limonene, and (**C**) L-Limonene.

Carveol significantly reduced the production of xanthomonadin at 0.4 mM and basal level of xanthomonadin was produced at ≥ 0.7 mM treatment (Fig. 4A). However, D-limonene and L-limonene treatments did not make significant changes in the production of xanthomonadin and the degree of change was also similar with two limonene isomers (Fig. 4B and 4C).

### Field-Emission-Scanning Electron Microscope (FE-SEM) Observation

We employed two different concentrations for the treatments of compounds in the *Xoo* culture prior to FE-SEM observation based on the results in Fig. 1. *Xoo* culture was treated with carveol at 0.7 mM of sub-minimal inhibitory concentration (MIC). Both D-limonene and L-limonene were used at 10 mM, the highest concentration employed in this study. *Xoo* cells treated with all three compounds showed similar ultrastructural changes with severe damages on their surface (Table 2). Specifically, many pore-like morphology was observed. The surface of the cells was wrinkled and shrunk with slight fold. Overall, the most cell population had severe morphological changes with the treatment of carveol and the less cells were damaged with the two limonene treatments. As a note, some cells with the treatment of L-limonene showed acute destruction in the morphology (Table 2).

### Real-Time PCR

**Fig. 5:**
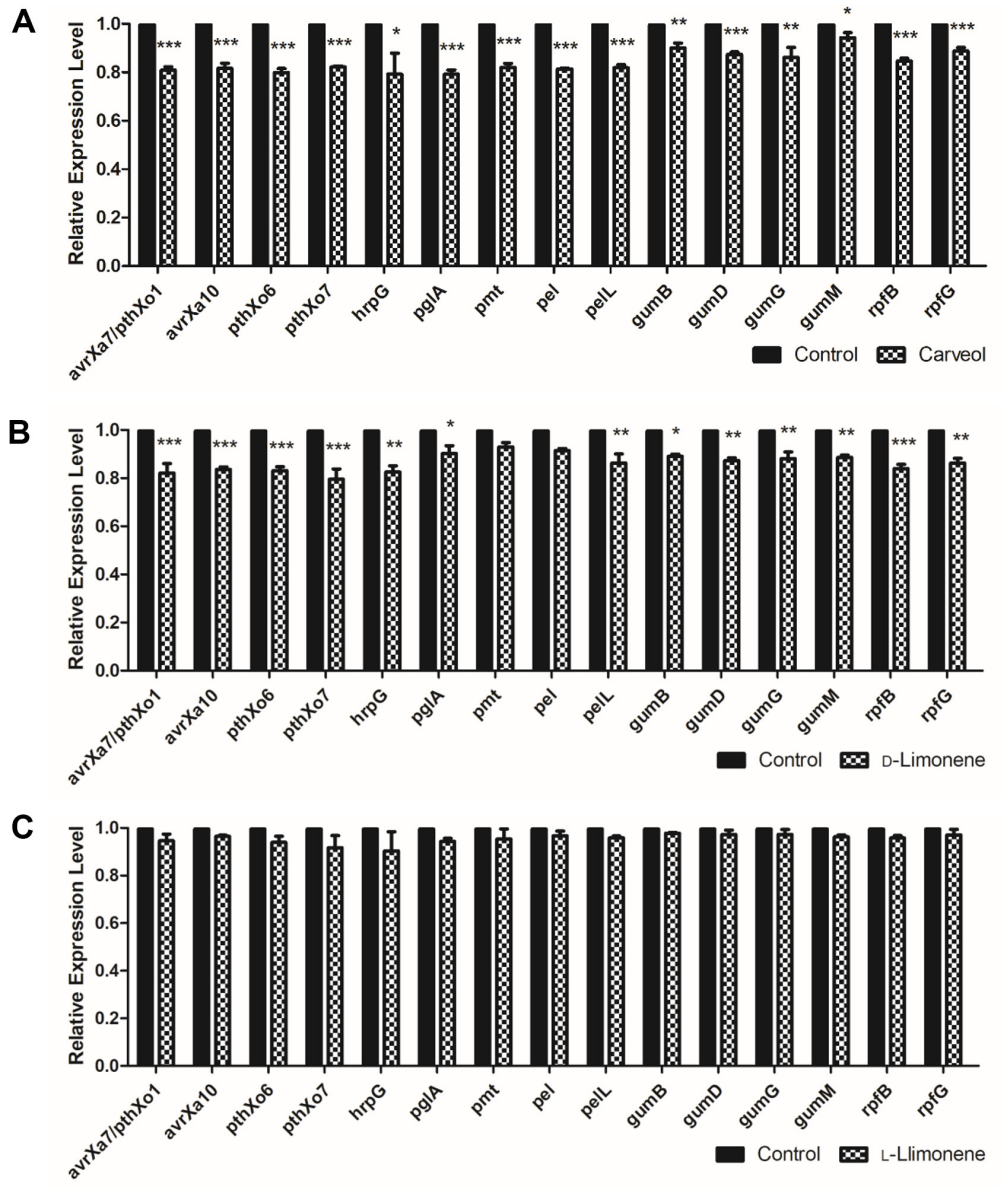
Quantification of relative expression level of virulence and biofilm gene using real-time PCR. This analysis was treated 0.7 mM carveol, 10 mM D-limonene, and 10 mM L-limonene. (**A**) Carveol, (**B**) D-Limonene, and (**C**) LLimonene.

The expression level of genes that are known for virulence and biofilm formation of *Xoo* was analyzed and compared after the treatment of carveol (0.7 mM). All genes except *gumM* employed were significantly down-regulated in their transcription level compared to that of control (Fig. 5A). In addition, D-limonene treatment (10 mM) decreased the relative expression levels of all target genes except *pmt* and *pel* compared to control levels but to a lesser extent (Fig. 5B). Unexpectedly, we did not find any significant changes in transcription levels of the target genes with the L-limonene (10 mM) treatment (Fig. 5C).

## Discussion

Plant natural products such as alkaloids, flavonoids, glycosides, terpenes, tannins, and polyphenols have potential as new agents to combat bacterial infections caused by various virulence factors. Phytochemicals interrupt QS by interfering with the signal molecules and receptors that cause bacterial QS [24, 25]. They inhibit biofilm formation by preventing the adhesion of bacteria and killing bacteria in mature biofilm [26]. Swarming and swimming ability of bacteria are restricted by reduced expression of the genes that are related with flagella and motility affected by a functionality of the natural products [27-29]. The plant phytochemicals inhibit the production of bacterial pigments and the activities of various enzymes (*e.g.*, sortases, proteases, coagulase, etc.) [25]. They also facilitate inhibition of the surfactants secreted by bacteria [30, 31]. The present study demonstrated the antibacterial effect of limonenes and its oxidation derivative. We revealed that carveol treatment significantly inhibited *Xoo* growth and the antimicrobial activity of D-limonene and L-limonene was to a lesser degree showing considerable suppression of *Xoo* growth at the highest concentration tested (10 mM). Therefore, we concluded that hydroxyl-containing carveol has higher antimicrobial activity than limonene isomers against *Xoo* growth. To examine minimal concentration of the isomers for complete inhibition of *Xoo* growth, further experiments should be performed with higher concentrations of D-limonene and L-limonene (> 10 mM).

Bacteria can swim in liquid and on solid surfaces through their flagella, which is the most common means of bacterial motility [32, 33]. Bacterial motility is an essential factor contributing to bacterial attachment and biofilm formation [34, 35]. Melatonin and thyme oil reduced *Xoo* motility [22, 36]. Microorganisms acquire resistance to antimicrobial agents through various methods, and biofilm formation is one such method. Limonene was confirmed to inhibit biofilm formation of *Bacillus cereus*, *E. coli*, and *Pichia anomala* [37]. Limonene has been demonstrated to be effective in inhibiting biofilm formation. In the present study, the crystal violet assay was performed to examine whether limonene isomers and carveol treatment can inhibit biofilm production. An assay using crystal violet as a proxy marker can quantify biofilm formation by using an indirect method [38]. Xanthomonadin is a yellow pigment found in a membrane to protect bacterial cells from photodamage and peroxidation maintaining a close relationship with the host plant [39]. Additionally, it has been reported that xanthomonadin is involved in biofilm formation [40]. Carveol-treated *Xoo* exhibited a decrease in motility, biofilm formation, and xanthomonadin production compared with the untreated *Xoo*. The results were in the same line with the antimicrobial activities of carveol shown in Fig. 1. Notably, carveol treatment resulted in dramatic decrease in the assays above mentioned, but both limonene isomers had activities to a lesser extent. Also, isomeric difference of limonenes did not confer any differential activities on motility, biofilm formation, and xanthomonadin production of *Xoo*.

Most antibiotic treatments cause destructive damages such as roughening, shriveling, shrinking, and holes in bacterial cell membranes [41, 42]. Similarly, it is reported that limonene treatment destroyed the cell membrane of multidrug resistant *E. coli* causing cell death [43]. Treatment of an antihelminth drug niclosamide changed *Xoo* into an irregular shape with hollow surfaces [21]. However, limonenes and carveol treatment in this study destroyed *Xoo* cell membranes causing devasting changes in the ultrastructual morphology.

The expression levels of virulence genes that induce *Xoo* to cause pathogenicity in rice and the genes involved in biofilm formation were quantified using real-time PCR. Among the virulence genes analyzed, *avrXa7* influences bacterial aggressiveness and disease symptom development and suppresses the hypersensitive response (HR). *avrXa10* inhibits the HR response. *pthXo1* induces the expression of *Os8N3*, a rice susceptibility gene. *pthXo6* targets the transcription factor *OsTFX1*. *pthXo7* influences on the bacterial growth and lesion length of *Xoo* with reduced virulence [3]. *hrpG* is essential for the expression of the *hrp* gene, which encodes the type III secretion system [11, 12]. *pglA* encodes polygalacturonase that degrades pectin [8]. The *gumBCDEFGHIJKLM* gene involved in biofilm formation regulates xanthan synthesis [10, 42-44]. *rpfG* is involved in QS and regulates virulence factors [45, 46]. Carveol and D-limonene downregulated the expression levels of genes analyzed in this study. Although there was a difference in the expression level between treated and untreated *Xoo*, the degree of decrease was not dramatic. Further study should consider higher concentrations of the compounds and the time points to be analyzed.

We here investigated the effects of limonenes and its oxidation derivative on *Xoo* growth. Carveol significantly affected the antibacterial activity, virulence, and biofilm formation of *Xoo* even at as low as 0.1 mM concentrations. It remarkably changed the morphology of *Xoo* cells and reduced the expression levels of virulence and biofilm formation-related genes. Although D-limonene and L-limonene exhibited a minor effect at the concentrations analyzed, we assumed that if higher concentrations are employed, the effect would be more definite to a greater extent. Together, the limonenes and carveol may be considered as an eco-friendly antimicrobial agent, with which we can reduce the use of synthetic chemicals that are harmful to human health and ecosystem. Formulations and relative compositions of the compounds should also be an interest to enhance the functionality.

## Figures and Tables

**Table 1:** Primer list used for real-time PCR.

Gene	Primer sequence		Reference
	Forword	Reverse	
*avrXa7* / *pthXo1*	GAGAGCATTGTTGCCCAGTT	CGGCGATTGATTCTTCTGAT	
*avrXa10*	CGTGATGTGGGAACAAGATG	GTGTTGTAGAGGGCGACGAT	
*pthXo6*	TCAGCAGCAGCAAGAGAAGA	CAACGCCCTGATTATGTCCT	This study
*pthXo7*	GGCTTGGTACAGCTCTTTCG	GTCTGATCTCCCTCGTGCAT	
*hrpG*	ACTCAGCGTTGGTCCATACC	TGCGCAGCTTGTAGATATGC	
*pglA*	TCGCTGGGTTCCAAGGCATT	GGCTTTGCGAACGAGACCTTT	
*pmt*	TGTTCGACAATGCCCTCATCC	GCCACAAAGCGGCTATTGATCG	[8]
*pel*	TGCGTTTCGGTAAGGTGCATCT	ACCGCTGATCTCGAACACATTACG	
*pelL*	GCGCATCGAAATGATCAACC	TCGTCCAGGCTGACGAAAT	
*gumB*	ATCGTGTTCCGCATGGTC	ATATCGCCGCCGTAAATCTC	
*gumD*	GCGTGCGATTCGTATGTTTC	GCGAGGAAGCTGTGTAAGT	
*gumG*	GCCAATGGTTCACGTCTGTA	GCACGCAAGGCAATGTAAG	[21]
*gumM*	TGTTGAGACGACGGGAATTG	CTGCCAACGCATGGAATAGA	
*rpfG*	GACGAGATGAGCGTGATGAA	GATAACCGGTGCCGTCATAA	
*rpfB*	ACGCGGTGAAGCGGGTCTTT	ATCACTGCGCTGCCGCTGTA	

**Table 2:** Morphology observation of *Xoo* treated without (control) or with compounds through FE-SEM.

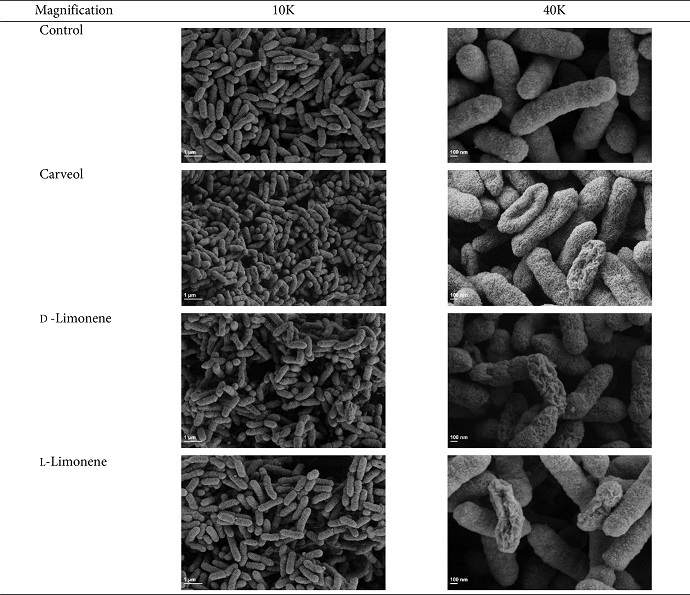

This analysis was treated 0.7 mM carveol, 10 mM D-limonene, and 10 mM L-limonene. 10K = 10,000, 40K = 40,000.
